# Supplementary material for: BAG2 promotes tumorigenesis through enhancing mutant p53 protein levels and function
Source: eLife. 2015 Aug 13;4:e08401. doi: 10.7554/eLife.08401 (PMC4561369; doi:10.7554/eLife.08401)
Supplement: Figure 4—source data 1. — DOI: http://dx.doi.org/10.7554/eLife.08401.014 [file elife08401s001.pdf]

Figure 4-source data 1. % of cells with different BAG2 localization in H1299 cells

	N	N>C	N=C	N<C	C
Vector					
Con	0.005±0.004	0.0154±0.009	0.033±0.006	0.791±0.01	0.156±0.02
mutp53 R175H	0.085±0.016	0.709±0.039	0.143±0.052	0.041±0.003	0.022±0.009
mutp53 R248W	0.144±0.012	0.701±0.067	0.125±0.061	0.025±0.014	0.006±0.008
mutp53 R273H	0.177±0.013	0.722±0.022	0.068±0.020	0.029±0.01	0.004±0.005
wtp53	0.007±0.006	0.020±0.013	0.032±0.020	0.77±0.031	0.172±0.021
mutp53 R175H-NLS	0.001±0.006	0.014±0.013	0.035±0.020	0.684±0.031	0.268±0.021

Note: Source data for Figure 4a. Data are presented as mean±SD.
