# Supplementary material for: BAG2 promotes tumorigenesis through enhancing mutant p53 protein levels and function
Source: eLife. 2015 Aug 13;4:e08401. doi: 10.7554/eLife.08401 (PMC4561369; doi:10.7554/eLife.08401)
Supplement: Figure 5—source data 1. — DOI: http://dx.doi.org/10.7554/eLife.08401.016 [file elife08401s002.pdf]

Figure 5-source data 1. % of apoptosis induced by 5-FU in Saos2 cells with and without ectopic expression of mutp53

	Con siRNA		BAG2 siRNA-1		BAG2 siRNA-2	
Time of 5-FU treatment (h)	0	48	0	48	0	48
Saos2-Con	6.235±3.48	60.49±6.61	9.925±3.909	66.05±2.022	14.35±3.733	65.84±8.074
Saos2-R175H	4.675±3.208	38.9±2.758	8.875±5.205	55.863±3.096	8.793±2.322	60.55±7.266
Saos2-R248W	7.88±0.543	31.373±2.747	13.933±2.759	52.633±1.601	13.033±4.554	64.99±2.319
Saos2-R273H	6.837±0.383	32.433±4.291	9±2.879	50.1±7.031	13.467±5.398	52.533±9.248

Note: Source data for Figure 5a. Data are presented as mean±SD.
