# Supplementary material for: BAG2 promotes tumorigenesis through enhancing mutant p53 protein levels and function
Source: eLife. 2015 Aug 13;4:e08401. doi: 10.7554/eLife.08401 (PMC4561369; doi:10.7554/eLife.08401)
Supplement: Figure 5—source data 2. — DOI: http://dx.doi.org/10.7554/eLife.08401.017 [file elife08401s003.pdf]

Figure 5-source data 2. % of apoptosis induced by 5-FU in HCT116 cells with and without mutp53

	Con siRNA			BAG2 siRNA-1			BAG2 siRNA-2		
Time of 5-FU treatment (h)	0	24	48	0	24	48	0	24	48
HCT116 p53-/-	9.367±1.554	58.367±4.805	82.2±1.076	18.033±0.759	61.783±3.792	92.283±0.558	17.867±0.382	64.883±4.17	92.5±1.532
HCT116 p53 R248W/-	8.367±0.621	39.517±4.781	64.15±0.721	14.05±0.173	55.7±2.656	88.65±0.568	14.85±2.97	62.393±1.508	89.667±2.627

Note: Source data for Figure 5c. Data are presented as mean±SD.
